# Nanocones: A Compressive Review of Their Electrochemical Synthesis and Applications

**DOI:** 10.3390/ma17133089

**Published:** 2024-06-24

**Authors:** Katarzyna Skibińska, Piotr Żabiński

**Affiliations:** Faculty of Non-Ferrous-Metals, AGH University of Krakow, al. Adama Mickiewicza 30, 30-059 Krakow, Poland; zabinski@agh.edu.pl

**Keywords:** nanomaterials, nanocones, electrodeposition, crystal modifier, template, catalysts

## Abstract

The development in the field of nanomaterials has resulted in the synthesis of various structures. Depending on their final applications, the desired composition and therefore alternate properties can be achieved. In electrochemistry, the fabrication of bulk films characterized by high catalytic performance is well-studied in the literature. However, decreasing the scale of materials to the nanoscale significantly increases the active surface area, which is crucial in electrocatalysis. In this work, a special focus is placed on the electrodeposition of nanocones and their application as catalysts in hydrogen evolution reactions. The main paths for their synthesis concern deposition into the templates and from electrolytes containing an addition of crystal modifier that are directly deposited on the substrate. Additionally, the fabrication of cones using other methods and their applications are briefly reviewed.

## 1. Introduction

The development of nanomaterials in the energy field is receiving more and more attention these days. By definition, nanomaterials have at least one dimension between 1 and 100 nm [1]. They can be divided depending on size, structural configuration [2], and application [3,4].

Nanocones are an example of one-dimensional (1D) nanomaterials that are characterized by two nanometric dimensions in three perpendicular directions [5]. They are usually synthesized using templates [6]. Other examples of 1D structures are, among other things, nanowires [7,8], nanorods [9,10], and nanotubes [11]. Their biggest advantages are their extreme surface–volume relationship and high surface area [12]. Therefore, they have found application in the energy conversion field, specifically in water-splitting systems [13,14]. Conical structures show some advantages over other 1D nanomaterials. They are more mechanically stable than nanowires, nanorods, and nanotubes. This means that free-standing cones do not tend to bend and fall like wires.

Electrodeposition is the “bottom-up” method used to synthesize nanostructures. This means that these structures are built from molecular species. Electrochemical techniques allow for the fabrication of nanomaterials with the desired morphology by changing the process parameters, e.g., temperature, applied current density, potential, electrolyte additives, and process duration. The produced nanostructures can be used in various fields, such as biotechnology [15,16], energy [17], and medicine [18,19].

A hydrogen evolution reaction (HER) is a step in H_2_ production through water electrocatalysis [20]. The important features in the catalyst’s design are its developed active surface area, high intrinsic activity, and fast transport of electrons [21]. The synthesis of the material on the nanoscale ensures a large active area. In this way, it is possible to limit the use of expensive metals from the platinum group [22,23].

Due to the constant focus on the nanomaterials’ applications as catalysts in HERs and the increase in the number of works on the synthesis of nanocones, this review gives a compressive summary of the achievements in this field until now. Special attention was paid to the electrochemical methods of the cones’ synthesis for the application in hydrogen evolution reactions.

## 2. Cones or Pyramids?

**Figure 1:**
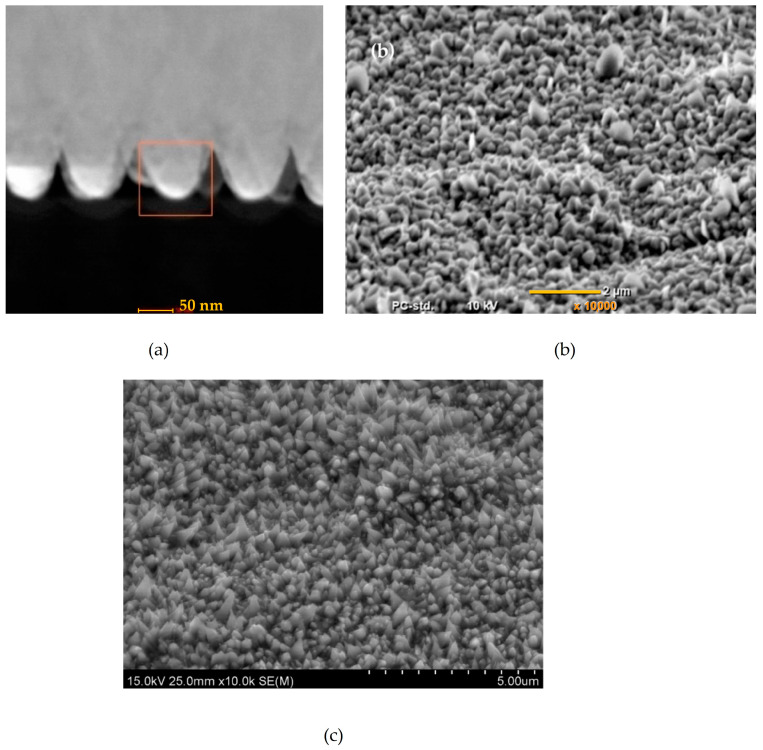
Shapes of (**a**) Co-Fe [26] (scale bar corresponds to 50 nm), (**b**) Co [27] (scale bar corresponds to 2 µm), and (**c**) Ni structures [28] (scale bar corresponds to 5 µm) which are called conical.

Reviews often pose this question when a paper on the nanoconical structures is submitted. In the literature, nanocones can be defined as carbon networks [24]. However, in this work, the term nanocones corresponds to the shape of the obtained structures. Until now, there are no requirements for their size and form. They can be round- or sharp-ended, with a round or square base, which explains the hesitations about the nomenclature. However, “cones” is preferentially used compared to “pyramids” [25]. These structures can grow in the direction perpendicular to the substrate or irregularly in all directions. Figure 1 shows examples of cone structures.

Conical structures became popular in electrochemistry due to their unique properties. The synthesis of the material in the form of nanocones increases the active surface area of the sample. The larger the area, the greater the number of catalytic sites available for the reaction, and consequently, an increased productivity of the catalysts [29]. Moreover, they often show superhydrophobicity [30], corrosion resistance [31], as well as high stability during the intensive hydrogen evolution [32].

Conical structures, depending on the materials used and desired properties, can be synthesized using many techniques, e.g., deposition in templates, electrochemical synthesis from solutions containing an addition of a crystal modifier, or laser ablation, usually combined with electrodeposition. In this review, the focus is placed on the electrochemical methods for these structures’ fabrication using one- and two-step methods. In this work, the terms “nanocones”, “conical structures”, “conically shaped structures”, and “pyramids” are used as synonymous.

## 3. Methods of Synthesis

### 3.1. One-Step Method

Nanocones are commonly fabricated by a single electrodeposition from an electrolyte solution containing the addition of chemical components. Thus, this method is called a ‘one-step’ method. This component, called a crystal modifier or capping agent, is added to the solution to block horizontal-to-surface growth and promote vertical growth. The mechanism of growth is driven by the screw dislocation. Initially, conical structures appear with screw dislocations in different directions, which eventually all become oriented in the same direction [33]. A template is unnecessary because anisotropy appears due to the growth kinetic differences along the different crystallographic directions [34]. It is also believed that the growth of Ni cones is related to the tip-discharge phenomenon [30]. Ni nuclei are mainly formed as 10 nm nanoparticles when the electrodeposition starts. Then, the larger nanoparticles, with the shape of pyramids, act as seeds for the nanocones’ growth. While these cones are becoming longer, smaller cones are hidden between them. Then, secondary nuclei can occur on the big cones. With more crystal modifiers added to the solution, sharper and higher cones can be obtained [35]. Moreover, the higher the current density, the smaller the apex angle of the nanocones [36]. The preparation of the substrate through etching and/or polishing can also influence the growth of cones [28].

A few chemical components are used as crystal modifiers, usually containing Cl^−^ ions. For example, ammonium chloride is unstable below 60 °C in nickel chloride solutions; therefore, the electrolyte must be heated. NH_4_Cl can change the preferred grain orientation of nickel coatings from (220) to (111) [37]. However, it acts like an inhibitor in the electrodeposition and delays the nucleation of Ni. In the case of CaCl_2_·2H_2_O, this reagent dissolves in the solution at low temperatures [38]. Thus, the solution can be stored at room temperature. However, when the solution’s temperature is about 60 °C, the apex angle decreases [38]. Ethylenediamine dihydrochloride (EDA·2HCl) is an expensive reagent that limits its use. On the other hand, NaCl is an easily available, inexpensive, and harmless chemical component. Boric acid is used as a buffer agent. However, it also acts as a capping agent [39]. E. Rahimi et al. investigated micro-nanocones deposition using an electrolyte solution containing NH_4_Cl and H_3_BO_3_ [40]. Using AFM, they were found to have about six steps due to the spiral growth of screw dislocation. The step heights of the two micro-cones were ~32 and ~45 nm, with terrace widths of ~58 and ~68 nm. Moreover, when the concentration of H_3_BO_3_ was sufficiently high, it enhanced the Ni nucleation process and improved deposition [41]. A literature review showed that the presence of Cl^−^ ions coming from NiCl_2_, from an electrolyte solution that also contained H_3_BO_3,_ was enough to obtain Ni cones [42]. Even a chemical such as Janus Green B can be a crystal modifier [43]. The higher its concentration, the more pyramids with smaller apex angles were deposited. L. Thang et al. synthesized Cu cones using pulse-reverse current (PRC) electrodeposition [44]. The growth driven by the screw dislocation probably originated from the high stress and supersaturation at 1 kHz. The authors did not discuss the influence of a small addition of NaCl (30 ppm) on the cone deposition. Z. Chen et al. [45] noticed that the application of pulsed electrodeposition to the synthesis of Ni cones resulted in the rapid formation of directionally ordered structures due to its influence on the nucleation and diffusion kinetics on the cathode. This method also requires the use of a lower content of NiCl_2_ than direct-current electrodeposition.

The materials synthesized in the form of cones using the one-step method are listed in Table 1.

To summarize, Ni cones are most often deposited by the one-step method using an electrolyte solution containing ammonium chloride as the crystal modifier. Conical Ni structures can be successfully used as a substrate for further synthesis. Thin layers of Cu were electrodeposited onto prepared Ni cones [57]. After annealing, Ni-Cu structures were obtained. Therefore, cones can be successfully used as a matrix. L.K. Wu et al. deposited Ni cones on Ni foam [58]. Then, this structure was immersed at 100 °C for 5 s in a solution containing Fe^3+^ ions. As a result, a bimetallic hydroxide layer on the Ni cones was obtained.

Ni cones were also synthesized from an electrolyte solution containing a crystal modifier on a picosecond laser-ablated micro-Cu surface [25]. Firstly, Cu foil was polished with SiC papers (grit 1500) and alumina powder (diameter 2.5 μm). The Cu microstructures were fabricated using a picosecond laser system. Then, the substrate was cleaned with ultrasound in acetone and electropolished. The activation process was performed in 10 wt.% HCl was at room temperature, and the sample was washed in distilled water afterwards. In the end, the deposition of Ni cones was performed. These structures showed superhydrophobic properties.

The production of cones is usually an antecedent step in synthesizing flower-like structures [59]. In this work, EDA was used as the crystal modifier, and H_3_BO_3_ was the buffer agent. Deposition for 400 s at 50 mA/cm^2^ allowed for the fabrication of many conical structures that grew into flower-like structures after the next 200 s. These deposits initially showed superhydrophilic properties, but after two weeks of storage in air, they turned superhydrophobic.

Nickel, iron, and cobalt are ferrous metals with ferromagnetic properties [60]. Therefore, their properties can vary when a magnetic field is applied. M. Huang et al. applied a magnetic field during the deposition of nickel cones from a solution containing NH_4_Cl as the crystal modifier [61]. Global flow was found to dominate in the setup compared to local flow [62]. It is believed that in a well-designed experiment, the applied magnetic field can support the growth of the conical structures.

The one-step method is simple and allows for the synthesis of conical structures in one electrodeposition process. However, controlling the structures’ shape, size, and orientation is complex.

### 3.2. Deposition in Prepared Substrates

Conical structures can be fabricated using pre-produced templates made of anodic aluminum oxide (AAO) or polycarbonates (PC). This technique requires the fabrication of a matrix and then the deposition of the material into its pores; thus, this approach can be called a ‘two-step’ method. The shape and size of the structures, and therefore, their properties, vary depending on the chosen template [63]. The anodization process allows for the fabrication of Al_2_O_3_ as parallel nanopores [64] that are perpendicular to the Al surface. The geometrical properties of hexagonal cells in AAO, i.e., pore diameter and height and the interpore distance, can be controlled by applying different process conditions like the electrolyte and its concentration [65,66,67], temperature [68,69,70], voltage [71,72,73], and duration of the anodization [74,75]. The two-step anodization process produces an AAO matrix with nanoconical pores. In the first step, a layer of alumina oxide is produced. Then, it is removed in a mixture of phosphoric and chromic acid. As a result, the pattern left on the surface is a starting point for the second anodization. The anodization conditions applied (electrolyte, temperature, and voltage) can be the same in both steps. However, the second step is usually much faster. To synthesize the conical template, cycles of fast anodization and the pore widening process in phosphoric acid [76] must be performed. In two-step anodization, the pore density can be controlled by changing the duration of the second-step anodization [73]. An example of the template fabrication with conical pores is as follows [76]: first, a long-step anodization is performed in 0.3 M oxalic acid solution at 45 V and 9 °C for 1 h. Then, the synthesized oxide layer is removed by immersion for 1 h in a mixed solution of 6 wt% H_3_PO_4_ and 1.8 wt% H_2_CrO_4_ at 60 °C. Then, the cycles of the alternating short-step anodization and pore-widening process are performed. In this step, the anodization is performed in 0.3 M oxalic acid solution at 9 °C. However, its duration is 25 s for the first cycle and 20 s for the others. The pore-widening process is carried out in 5 vol% H_3_PO_4_ solution at 30 °C for 720 s. By changing the number of cycles, the ratio of the height of conical pores to their diameter can be controlled. It is important to prepare the surface of the Al foil before the anodization process by electropolishing. In the literature, the term “multistep conical nanowires” appears [77] as well. They can be obtained by changing the composition of the electrolyte solution.

Matrixes fabricated using polycarbonate foils are usually called membranes and can be produced using ion tracking. This method can be combined with electrodeposition [78]. The geometry of pores depends on the etch rate ratio along the track to the etch rate of the undamaged bulk material [79]. Polycarbonate foils are irradiated with heavy ions, resulting in homogenous etching. Because polymers are usually insulators, the energy loss by a charged particle results in a loosely bound material called nuclear tracks [80]. After irradiation with ions, chemical etching creates pores in this bound material, which converts tracks into pores. The etchant concentration, temperature, and applied voltage influence the etching rate [81]. This method allows for the synthesis of narrow and long pores in membranes. The structures synthesized in PC membranes show homogeneity over 1 cm^2^ [81]. These templates are mostly used to synthesize nanowires [82,83,84]. Three-dimensional (3D) networks made of interconnected nanowires are receiving more and more attention [85]. An example of these membranes’ preparation procedure from [78] is as follows: the PC foil is irritated with Bi (under a normal beam incidence with 9.5 MeV/u) and U (under a normal beam incidence with 11.1 MeV/u) ions. Then, the ion tracks are selectively etched using 9 M NaOH and methanol from one side.

Examples of synthesized conical structures using templates are listed in Table 2.

The synthesis of conical structures using AAO templates is rarely performed due to the limited mechanical stability of these materials. Alumina oxide is an insulator. Therefore, conductivity must be provided to fabricate cones of the desired metal or alloys using electrodeposition. T. Nagura et al. first deposited Pd particles into an AAO template, which catalyzed Ni deposition [86]. Before the electrodeposition of Cu [76], a thin layer of the same metal was sputtered to ensure the conductivity for the deposition. The synthesis of alloys in an AAO matrix is also possible [26]. However, this sputtering can mask the sharp-ended tip of the conical nanopore of the template. The obtained nanocones, thanks to the uniformity of the used template, are homogenous structures with heights below 150 nm. Therefore, the development of an active surface area is significant when the materials are fabricated in this shape. Examples of anodizing conditions are listed in Table 3.

The anodization conditions necessary to obtain conical nanopores are similar, with the voltage and temperature range considered independently from the solution used.

In the case of PC membranes, the diameter and depth of the pores linearly increases with the etching time [91]. Moreover, the higher the temperature, the faster the etching process. These cones are usually micro-sized. Depending on the material deposited in templates, e.g., dichloro-methane or *N*-methyl-2-pyrrolidone (PC), NaOH (AAO) and H_3_PO_4_ (AAO) can be used to remove the matrix.

The matrix has many advantages. It allows the size and shape of pores to be controlled by adjusting the process parameters. Due to its simplicity, researchers can successfully use this method at universities and in industry. One of the disadvantages of deposition in templates is the integration of free-standing structures in the desired system. F. Roustaie et al. proposed a new approach [92]. They fixed the PC membrane with conical pores on a gilded glass wafer. Then, the metal was electrodeposited through small tips.

### 3.3. Other Methods

These structures can also be deposited on a previously prepared surface. Ni pyramids were produced by electrodeposition on Cu micro-structures obtained using an ultra-fast picosecond laser system [25]. Before the electrodeposition of Ni, Cu deposits were electropolished in a mixture of 70 g/L Na_2_CO_3_, 10 g/L KOH, and 10 g/L sodium dodecyl sulfate (SDS) and activated in 10 wt % HCl. The samples obtained in this way showed long-term superhydrophobic properties. Conical structures were also grown by promoting the formation of well-aligned polypyrrole nanostructures using hydrogen bonding from a phosphate buffer solution (PBS) [93]. Due to the high concentration of pyrrole, the steric hindrance effect appeared and boosted the formation of conically shaped 3D structures. Then, a thin layer of RuO_2_ was deposited using sputtering to obtain a supercapacitor. H.S. Maharana et al. obtained Cu-ZrO_2_ through pulsed electrodeposition using an electrolyte solution containing different concentrations of cetrimonium bromide [94]. When the concentration was 0.5 g/L, standing hemispherical morphologies with vertically aligned nanocone arrays were fabricated. The coatings showed superhydrophobic properties when the added amount was 0.5 or 1 g/L. Micro-cones of TiO_2_ were obtained by combining laser ablation and a hydrothermal treatment. By changing the hydrothermal temperature, the morphology of secondary nanostructures on the surface of micro-cones varied from flocs to filamentous [95]. Also, conical ZnO structures can be obtained using the hydrothermal method [96].

**Figure 2:**
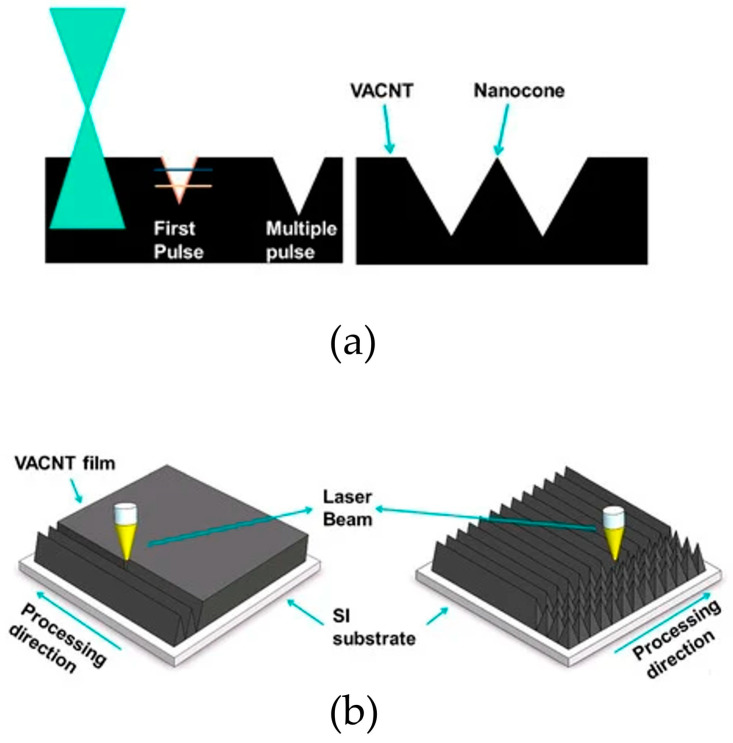
Example of fabrication of (**a**) single cone and (**b**) arrays of cones using a laser beam [97].

Laser ablation is another method used to fabricate cones [97]. Figure 2a shows the triangle shape left after vertically aligned carbon nanotubes (VACNs) are removed with the first and multiple pulses. An energy greater than the damage threshold must be used. With continuous transverse and longitudinal scans, nanocone-shaped carbon nanotubes can be synthesized (Figure 2b).

Conical structures can occur in poly(ethylene terephthalate) (PET) and polyimide (PI), which are strongly absorbing polymers [98]. Often, post-ablation arrays of cones are visible on a target surface, like MgB_2_, and they can be explained as inhomogeneities of the surface resistant to laser ablation that survived in the form of the cone’s tips [99].

**Figure 3:**
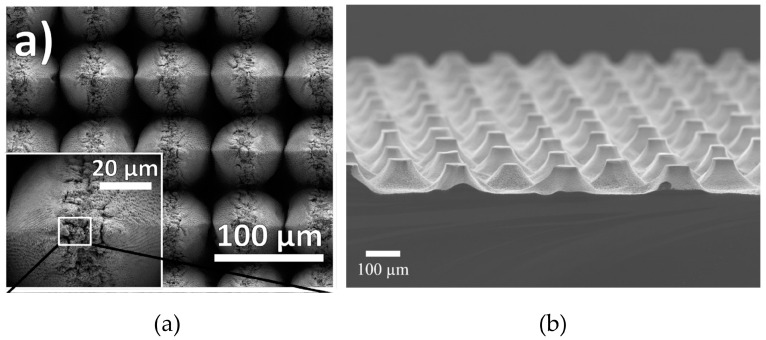
SEM images of (**a**) Cu pyramids [100] and (**b**) Si cones [101].

Cu pyramids were obtained using the femtosecond laser micromachining process (Figure 3a) [100]. In this method, the laser beam is focused by lenses on the substrate surface. The Cu structures, shown in Figure 3, were synthesized using a Ti:sapphire laser with a <100 fs pulse duration, 800 nm wavelength, and 10 kHz repetition rate.

Moreover, metal-assisted chemical etching (MACE) can be used to fabricate Si conical structures [101]. This complex technique consists of the following steps: (a) photolithography, where the photoresist is a masking layer for the metal deposition; (b) chemical deposition of Ag particles for 90 s; (c) MACE etching in HF, H_2_O_2_, and deionized water (DI), in a ratio of 4:7:40 *v*/*v* at 30 °C for 5 h; (d) dry oxidation at 850 °C under an O_2_ flux for 3 h; and (e) dissolution of oxide in mixture of HF and H_2_O in a ratio of 1:9 *v*/*v* for 60 s. The whole procedure allows for the synthesis of the structures shown in Figure 3b.

Figure 1 and Figure 3 show that the shape and size of the conical structures vary depending on the synthesis method and process parameters. Pyramids were synthesized on nickel using stationary ablation [102]. During the ablation, nanoparticles were created. They were attached to conical structures. If the sample was in motion during the process, multiple nano-particle coatings on the cones were present. If the ablation was stationary, only a single shell of nanoparticles was observed.

## 4. Applications

Due to nanocones’ unique properties of a high active area-to-volume ratio and increased catalytic properties, conical structures are commonly tested in hydrogen evolution reactions.

The reaction of hydrogen molecules created on the electrode involves two moles or electrons per mole of products.

When the environment is acidic, the H_3_O^+^ ion is the reacting substrate at E_0_ = 0 V (NHE, normal hydrogen electrode):

(1) $$2H^{+}+2\bar{e}\;=\;H_{2}$$

In an alkaline environment, the molecule H_2_O is the substrate of the hydrogen reaction at E_0_ = −0.828 V (NHE):

(2) $$2H_{2}O+2\bar{e}\;=\;H_{2}\;+\;2{OH}^{-}$$

when Reactions (1) and (2) take place, the electrolyte around the cathode alkalizes. These reactions are also characterized by a high degree of reversibility, which is especially observed in metals from the platinum group. Due to the increased active surface area and enhanced catalytic performance compared with flat bulk coatings, conical structures are commonly tested as catalysts in HERs.

The detachment of hydrogen bubbles from conical surfaces and an unmodified Ni foil was investigated by Q. Ren et al. [47]. They noticed that the bubbles detached faster and were smaller on cones. Moreover, conical samples achieved a high current density at a lower overpotential than the Ni foil. Furthermore, the device fabricated with Ni cones on a 3D-printed lattice showed more than 95% performance after 100 h of the water-splitting reaction at room temperature. Nickel conical structures showed a lower hydrogen reduction onset potential and a 10 times higher exchanged current density than the nickel film [103]. Thus, the synthesis of metals and alloys in the form of cones enhances the catalytic activity of the coatings.

**Figure 4:**
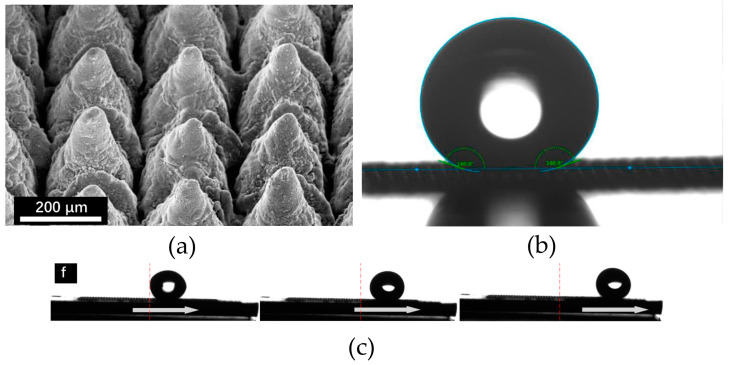
(**a**) Example of scanning electron microscopy (SEM) image of the as-prepared surface (magnification: 100×); (**b**) wettability measurement; (**c**) water droplets sliding off the surface. Images taken from [95].

L. Krause et al. compared the catalytic activity of three different samples, focusing on their roughness, wettability, and active surface area [104]. It turned out that more hydrophobic samples had worse performance at industrially relevant current densities. Larger hydrogen bubbles detached from the samples with a higher hydrophobicity. Therefore, due to the electrode surface area blockage by H_2_, the influence of the nanostructuring was irrelevant. Wettability is another crucial factor to consider during the catalysts’ evaluation. It can be defined as the balance of gas–liquid–solid on the electrode surface [105]. In the case of conical structures, they also show superhydrophobic properties [49]. The contact angle value between the solid/liquid interface and liquid/gas interface through the liquid phase is more than 150°. This ability relates to good anti-icing properties. Micro-cones of TiO_2_ show the ability to delay the freezing of water droplets [95]. These structures are shown in Figure 4.

In this work, all micro-cones (Figure 4a) synthesized using laser ablation and hydrothermal treatment showed a contact angle greater than 160° (Figure 4b). Moreover, the surface was slippery. The water droplets just slid off the surface, as shown in Figure 4c. Unfortunately, superhydrophobic coatings usually show poor stability [106].

The properties of the conical structures can be tuned after their synthesis using various approaches. A thin layer of Rh was electroless deposited on Ni cones [107]. After just 10 s of galvanic displacement, the structures showed a higher catalytic performance and corrosion resistance in 1 M NaOH. The wettability of the surface strongly depends on its composition. The surface of Co-Ni conical structures was oxidized and reduced in a furnace [55]. With the changes in oxide content, the surface’s wettability varied from hydrophilic to hydrophobic when the oxide content was low and high, respectively. In this work, as well as in [59], the authors highlighted the importance of sample storage. After two weeks of exposure to air, the sample turned from superhydrophilic to superhydrophobic. This phenomenon relates to the nanostructured surface and the adsorption of air-borne hydrocarbons, which reduce the surface free energy. The conditions under which catalysts are tested are usually far from those that ensure scalability and the possibility of their application in industry [22].

An important factor that allows for the comparison of catalytic performances is the active surface area, which corresponds to the area where the electrochemical reaction occurs. Therefore, it is an intrinsic property which must be determined. A literature review showed that synthesizing conical structures creates a larger active surface area compared with that of the bulk material. The Electrochemical Active Surface Area (ECSA) of Ni cones, determined using Double-Layer Capacitance measurements, is about 2.8–3.4 times higher than bulk Ni [104]. The geometric surface of both samples was the same.

Many methods can be applied, e.g., measurements of Double-Layer Capacitance (DLC) by Cyclic Voltammetry (CV) or Electro-Chemical Impedance Spectroscopy (EIS), the Brunauer–Emmett–Teller method (BET), and Atomic Force Microscopy (AFM). However, their results cannot be easily compared [108]. Using AFM, some assumptions on the difference in surface development should be made. This method does not provide any information about the real active surface area. Using the BET technique [109], the N_2_ gas is assumed to have access to the entire surface and is adsorbed in infinite layers with no interlayer interaction. Using this method, the sum of active and inactive areas is provided. In the case of DLC measurements with CV, the ECSA is determined using the changes in DLC with the scan rates. This method shows large errors due to its approximate character because the capacitance of an ideal flat catalyst surface (C = 0.04 mF/cm^2^) is often assumed to be the same for many materials. This capacitance can also be determined using EIS. The observed response is fitted to this measurement’s corresponding equivalent electric circuit (EEC).

The active surface area is crucial; therefore, researchers are working on developing approaches for its accurate determination. Authors have proposed a protocol for determining the ESCA of nonmetallic catalysts based on EIS measurements [110]. The ECSA of Ni samples was determined using EIS [111]. Different results can be obtained depending on the chosen approach.

Hydrogen peroxide is a crucial chemical that is commonly in used water treatment [112,113] and chemical synthesis [114]. Its residuals can be found after sterilization and packaging processes in the food and beverage industry. It is also present in the human body. However, there is a limit above which hydrogen peroxide is toxic to humans, especially if ingested or inhaled [115,116]. Synthesized cones can be successfully applied as sensors for the reduction of H_2_O_2_. A literature review showed that using the one-step method instead of synthesis in templates ensures the fabrication of less active but more stable structures [32]. Their Limit of Detection (LOD) and Limit of Quantitation (LOQ) were 0.18 mM and 0.62 mM [76]. Moreover, nanocones can be used as a gas sensor for formaldehyde [96].

A literature review showed that conically shaped structures show various properties and, therefore, can be applied in other fields. Nanoconical 3D a-Si and polydimethylsiloxane structures show interesting optical anti-reflection properties and can be used in photonics [117,118]. Conical Co microstructures can be applied in low-temperature solid-state bonding [53]. By changing the process parameters, the size of the cones can be controlled. When the cones’ height and diameters are ~610 nm and ~490 nm, respectively, a seamless bonding interface at 190 °C is achieved. Additionally, these conical structures showed a high hardness of 5.28 GPa. Furthermore, after 81 h of oxidation at 190 °C, the bonding strength was still higher than 40 MPa. Also, Cu cones can be applied in low-temperature bonding [52]. Ni-modified boron nitride nanocones can be applied as nonlinear optical active drug carriers [119]. Conical carbon structures can be used in various applications, e.g., NH_3_ detection [120] and phononic devices as thermal rectifiers [121]. Furthermore, conical structures are promising materials in cancer diagnosis [122]. Moreover, literature reviews showed the possible application of conically shaped materials in solar cells [123,124,125,126]. F. Sobhani et al. used conical metallic nanoparticles in simulations of silicon solar cells [127]. Their presence enhanced the cells’ photocurrent.

## 5. Concerns and Perspectives

One of the biggest concerns about the deposition of conical structures using the one-step method is its limitations. As noticed in Table 1, few metals have been electrodeposited in the form of cones. Due to the restricted size of crystal modifier ions used, few new conical materials can be fabricated using this approach. This problem could be solved by choosing the appropriate chemical additives and process conditions. This is especially important since this method’s low cost and simplicity make it available for use in research and development facilities. Moreover, fabricated cones are often characterized by a non-uniform size and direction of growth. This aspect is crucial in the estimation of the real active surface area. These problems can be solved using specific deposition techniques, e.g., pulse-reverse current (PRC) electrodeposition. Additionally, the substrate can be prepared by patterning using lithography [128]. The one-step method is an inexpensive technique that can be successfully scaled. It does not produce additional waste compared with the common deposition method for Ni coatings. The required temperatures are also low.

In the case of deposition in pre-prepared templates, the fabricated structures show insufficient resistivity to the intensive evolution of hydrogen [32]. Foils of free-standing nanocones were destroyed during HERs. Even prolonging the electrodeposition process and increasing the film thickness did not enhance its stability. The use of a matrix requires their purchase or preparation. Their production is energy- and time-consuming. Templates offered by the companies are easily accessible. However, their quality may be low depending on the price and producer. Moreover, the available commercial matrixes allow for the synthesis of nanowires but not nanocones. So, the independent synthesis of these templates in the lab is required. The fabrication of the templates and further deposition of metals and alloys can be performed at the industrial scale. However, this method is time- and energy-consuming. Moreover, toxic chromic acid is used. Therefore, choosing this technique to synthesize catalysts with low stability seems unreasonable.

Other methods are probably not popular due to the simplicity and low-cost of the one-step method and deposition in template method. Moreover, they usually allow for the synthesis of 3D structures instead of 1D structures.

Finally, the analysis of nanomaterials usually requires high-resolution devices, e.g., transmission electron microscopes with a Focused Ion Beam (FIB) system, X-ray photoelectron spectroscopy (XPS) system, and atomic force microscopes. These methods are not easily accessible to many scientists in academia and industry. It creates problems in understanding and proper interpretation of results. However, with the development of technology, these types of equipment are becoming less expensive, occupy less space, and can be achievable for more research groups. Moreover, the mentioned active surface area cannot be easily determined, which is a real obstacle compared to the synthesized catalysts. Apart from methods requiring complex devices, wettability measurements are an important factor that is usually connected with the significant changes in the catalytic activity of the conical samples. Then, a simple contour analysis can be applied to determine the contact angle values.

## 6. Conclusions

This work is a detailed review of the synthesis and applications of nanocones, with a special focus on electrochemical fabrication methods. Due to their special properties, wettability, mechanical and corrosion resistance, and nanometric size, they can be successfully applied in catalysis. However, some concerns must be addressed in the further development of these methods when the synthesis of new materials is desired. Due to the lack of defined requirements for shape and size, the conical structures can differ. Still, new works have appeared considering the synthesis or application of conical structures. Therefore, this topic and the enormous development in nanomaterials and renewable energy will continue to be investigated.

## Figures and Tables

**Table 1:** Literature on cones synthesized using the one-step method.

Material	Crystal Modifier	Addition of Crystal Modifier		Duration [s]	Current Density [mA/cm^2^]	Reference
		[g/L]	[M]			
Ni	NH_4_Cl	0–40	-	60–1500	10–40	[42]
		40	-	-	-	[46]
		-	1.4	480	20	[47]
		10–40	-	600	10–40	[48]
	(NH₄)₂SO₄	35	-	300–2100	15	[49]
	EDA·2HCl	200	-	112–900	20	[36]
	EDA·2HCl	-	0–2.115	240 and 480	20	[50]
	EDA·2HCl	-	1.5	660	15	[51]
	CaCl_2_·2H_2_O	-	0–2.4	120–900	10–30	[38]
	NaCl	-	0–4	120–1200	10–40	[35]
	H_3_BO_3_	-	0–2	1800–18,000	1–30	[39]
Cu	NaCl	3·10^−5^	-	60–1800	100 *	[44]
	NaCl	0–9·10^−5^	-	-	4 **	[52]
	Janus Green B (JGB)	0–0.2	-	30–300	12	[43]
Co	NH_4_Cl	100	-	10–1200	20–350	[27]
	NH^4+^ or -NH_2_	-	-	40–80	100	[53]
	NH_4_^+^	0–200	-	40	100	[54]
Co-Ni	NH_4_Cl	100	-	40	350	[55]
Co-Fe	NH_4_Cl	40 and 100	-	300–1200	20	[56]

* average current density. ** peak current density, 20 ms on and 1 s off for 2000 cycles.

**Table 2:** Nanocones synthesized in prepared templates.

Material	Template	Diameter Base/Height [nm]	Reference
Cu	AAO	62.3–104.9/133.5–151.8	[76]
Ni	AAO	100/100	[86]
		100/100	[87]
		100/100–500	[88]
Ni, Cu, Fe	AAO	-/576	[89]
Co-Fe	AAO	110.4/73.5	[26]
Cu	PC	1470/28,000	[78]
Au	PC	1000–8000/1000–11,000	[90]
Pt	PC	70–1500/700–1100	[91]
Ni	PC	7000/-	[92]

AAO—anodic alumina oxide; PC—polycarbonates.

**Table 3:** First (long) and second (short) step anodization condition examples.

First Step				Second Step				Reference
Solution	Voltage [V]	Duration [min]	Temperature [°C]	Solution	Voltage [V]	Duration [s]	Temperature [°C]	
0.3 M H_2_C_2_O_4_	45	60	9	0.3 M H_2_C_2_O_4_	45	25 * and 20 **	9	[76]
	40	600	16		40			[86]
0.3 M C_6_H_8_O_7_	40	-	16	0.3 M C_6_H_8_O_7_	40			[87]
0.3 M H_2_C_2_O_4_	45	60	2	0.3 M H_2_C_2_O_4_	45			[26]

* first cycle. ** subsequent cycles.

## Data Availability

All data are contained within the article.
